# Angstrom-Scale Water Layer Structure on van der Waals Materials Probed by 3D Atomic Force Microscopy: From Acidic to Alkaline Aqueous Solutions

**DOI:** 10.1021/acs.langmuir.5c05798

**Published:** 2026-02-10

**Authors:** Zhen Tang, Ricardo Garcia

**Affiliations:** Instituto de Ciencia de Materiales de Madrid, CSIC, 28049 Madrid, Spain

## Abstract

The structure of interfacial water governs the reactivity and surface properties of solid surfaces. Yet a molecular-scale understanding of how the concentration of H^+^ or OH^–^ species affects the interfacial hydration structure remains elusive. Here, we use three-dimensional atomic force microscopy (3D-AFM) to map with angstrom-scale resolution the interfacial liquid layer structure on mildly hydrophobic van der Waals (vdW) materials as a function of the pH. The liquid layer structure of MoS_2_ and graphite surfaces revealed the presence of 2–3 hydrocarbon layers separated by ∼0.45 nm. The pH value neither prevented the presence of hydrocarbon layers nor facilitated the removal of hydrocarbon layers from graphite or MoS_2_ surfaces. In contrast, the interfacial layer structure on a hydrophilic surface (mica) revealed the formation of 2–3 hydration layers separated by ∼0.28 nm for both acidic and basic conditions. A theoretical model predicted the formation of hydrocarbon layers on van der Waals materials in the presence of trace amounts of hydrocarbons (∼15 μg/m^3^). We propose that hydrocarbon layers are indispensable to explain the properties of van der Waals material–water interfaces.

## Graphical abstract

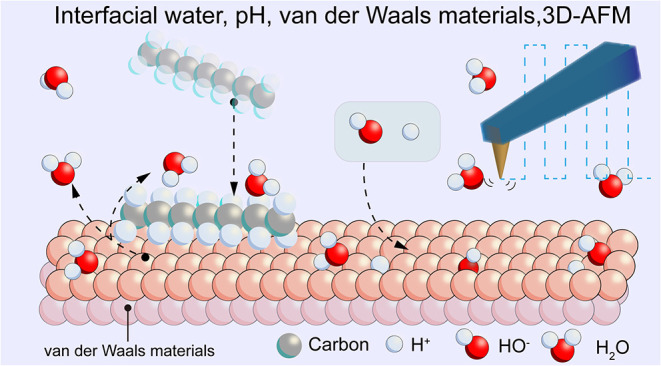

## 1 Introduction

The pH measures the concentration of protons in an aqueous solution. The pH is a fundamental parameter that has large implications in chemistry, biology, and materials science. However, molecular-scale resolution experiments aimed to study how the concentration H^+^ or OH^–^ species affects the interfacial water layer structure are missing.

The influence of pH on the interfacial water structure might have fundamental and practical implications. For example, the hydrogen evolution reaction rate depends strongly on the pH.^1,2^ In molecular biology, the pH is a key parameter to control the stability of biological structures such as collagen nanoribbons.^3^ Vibrational spectroscopy measurements performed on silica surfaces showed that cations could modify the solvent behavior at basic pH values.^4^

The interfacial water layer structure for graphite and MoS_2_ crystals as a function of pH has never been measured. Those materials exhibit several singular mechanical, electronic, and tribological properties which make them suitable for many applications ranging from energy storage,^5^ water filtration,^6^ and catalysis^7^ to lubrication.^8^ Some of those applications involve direct interaction of the solid surface with aqueous solutions. For example, MoS_2_ has emerged as a promising electrocatalysis candidate for the hydrogen evolution reaction,^7^ while graphite is widely used as an electrode in energy storage applications.^9,10^

It is well-known that graphite, 2D materials, and van der Waals (vdW) crystals are prone to incorporate hydrocarbon molecules from the environment.^11–13^ In particular, straight-chain alkanes might spontaneously form stripe-like patterns on graphene,^14–16^ graphite,^17–21^ MoS_2_,^22^ and h-BN^23^ surfaces.^14–20^ In fact, the process is so pervasive that it is extremely hard to keep them under pristine conditions.^11^ In aqueous solutions, hydrocarbons might displace the water molecules from the interface to give rise to a water-depleted region.^24^

The presence of hydrocarbon layers on van der Waals crystals might modify the wettability, catalytic activity, and dielectric properties of the material–water interface. It has been argued that organic impurities might limit the capacitance of carbon-based supercapacitors.^25^ In fact, the etching-resistant features of monolayer transition metal dichalcogenides grown on hydrophobic surfaces^26^ could be explained by a passivation effect associated with the formation of hydrocarbon layers. At a fundamental level, the interpretation of the dielectric permittivity of nanoconfined water in terms of the restricted dipole orientations^27,28^ might be challenged by the presence of hydrocarbon layers.

Recently, it has been shown that the application of a negative surface potential or a cyclic voltammogram might remove the organic contaminants from MoS_2_ ^29^ or graphite interfaces.^30^ The protocol involved the use of an electrochemical cell, which might preclude many applications. In addition, the application of a surface potential might modify the surface. Those results provided an additional motivation to study whether the pH of the surrounding solution could reorganize the interfacial water layer structure by preventing the formation of hydrocarbon layers on van der Waals crystals.

Three-dimensional atomic force microscopy (3D-AFM) has emerged as the most powerful method to resolve interfacial liquid structures with atomic or molecular-scale resolution in the three spatial coordinates.^31–36^ Three-dimensional AFM has been applied to study the effect of electrolytes on the interfacial hydration structure.^36–42^ To our knowledge, the influence of the pH value on the hydration layer structure has never been studied by 3D-AFM. In fact, very few AFM experiments have been performed to study the influence of the pH on the surface structures.^43,44^

Here, 3D-AFM is applied to study with molecular-scale resolution the interfacial layer structure on graphite and MoS_2_ as a function of the pH. The experimental study included a range of pH values from very acidic (pH 1) to very basic (pH 13) solutions. On mica, a prototypical hydrophilic surface, the interlayer distance was 0.29 nm. This value is very close to the average separation among water molecules in bulk water (∼0.28 nm). In contrast, on graphite and MoS_2_ (under standard working conditions), the interlayer distance value was 0.44 nm. Those values were very close to the nominal diameter of a straight-chain alkane molecule (∼0.42 nm). 3D-AFM images showed that the interlayer liquid layer structure of crystalline surfaces was hardly affected by the pH. In fact, the adsorption of alkanes and the formation of hydrocarbon layers on pristine-prepared MoS_2_ and graphite surfaces could not be prevented by the concentration of H^+^ or OH^–^ species of the solution. A theoretical model based on free energy considerations supported the formation of hydrocarbon layers on MoS_2_ surfaces from trace amounts of alkane molecules. Based on those results, we propose that hydrocarbon layers should be explicitly considered to explain the properties of 2D materials and van der Waals–water interfaces.

## 2 Results and Discussion

Liquids tend to form periodic solvation layers near a solid surface.^11^ As a consequence, the particle density oscillates near the solid interface (Figure 1a). Imaging and characterization of solvation layers at the molecular scale are readily provided by 3D-AFM. Hydration layers are separated by about 0.30 nm. If the separation between layers is close to or above the diameter of a straight-chain alkane molecule (∼0.42 nm), the layers are called hydrocarbon layers.

**Figure 1:**
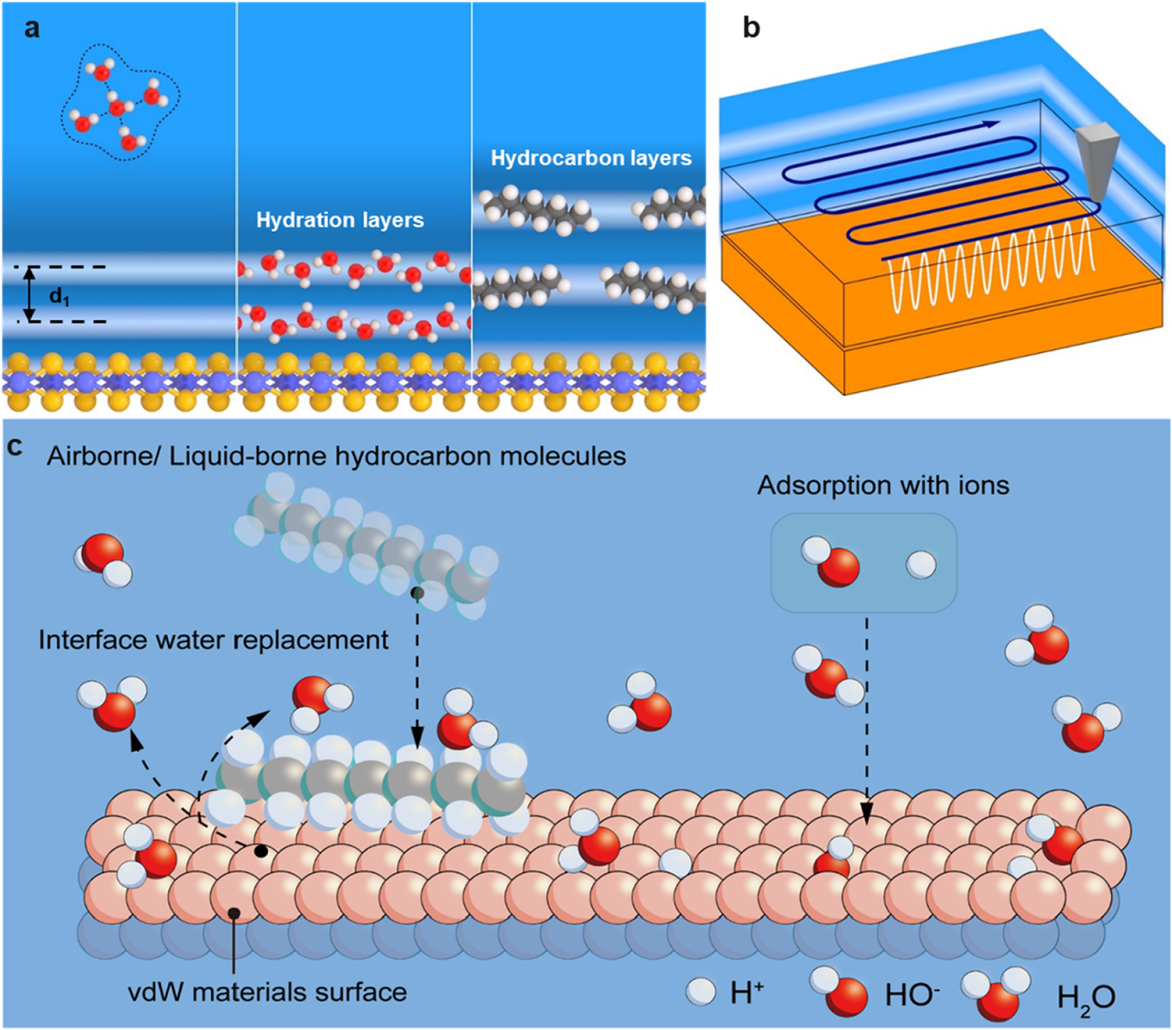
(a) Out-of-plane (*xz*) profiles of the liquid density near a solid surface. Definition of the hydration and hydrocarbon layers. Far from the surface, the water adopts a local tetrahedral configuration (bulk water). (b) Schematic diagram of 3D-AFM operation. (c) Schematic diagram of a van der Waals material–water interface. The adsorption of hydrocarbons on van der Waals surfaces immersed in water under standard working conditions is unavoidable. Atom sizes are not drawn to scale.

Figure 1b shows a schematic diagram of a 3D-AFM mapping experiment. The AFM tip images the solid–liquid interface by acquiring a force–distance curve at each *xy* position. For a fixed *y*_*i*_ position, those curves are combined to generate the force map *F*_*yi*_ (*x*, *z*). Those maps are grouped to generate a volume map of the interface. The features observed in a *F* (*x_i_*, *y_j_*, *z*) curve have been linked to the particle density profile of the liquid near the solid surface.^45–48^

Figure 1c provides a schematic diagram of a van der Waals material–water interface. The adsorption of hydrocarbons and the subsequent formation of stripe-like patterns is a common feature of van der Waals material–water interfaces. They have been observed on graphene,^15,18,21^ graphite,^15,18,23^ MoS_2_,^22^ and h-BN^23^ surfaces. A large body of experimental and simulation results^11,22,23^ backed by correlative 3D-AFM and Raman spectroscopy experiments^30^ demonstrated that the molecular species forming the interfacial layers observed on vdW material–water interfaces were airborne or liquidborne straight-chain hydrocarbons.^11,22,23^ Those hydrocarbons displaced interfacial water molecules and gave rise to an interfacial layer structure different from that associated with hydration layers.

### 2.1 Influence of the pH on the Interfacial Layer Structure: Mica, Graphite, and MoS_2_

The concentration of protons in a liquid (pH value) is a key parameter in characterizing an aqueous solution. The pH controls a variety of chemical and biological processes. Its value in a solution can be measured and modified with ease. Therefore, it might offer a convenient way to tune the interfacial layer structure of solid–water interfaces.

Muscovite mica is a hydrophilic aluminosilicate whose lattice substitutions confer a stable negative surface charge. Its surface is commonly used as a model system to understand how water interacts with a solid surface.^49,50^ Freshly cleaved mica terraces are atomically flat and free from airborne hydrocarbons. This system provides an ideal platform to reveal the key features of the hydration layer structure. The two-dimensional force maps (Figure 2) revealed a layered structure characterized by alternating high (light) and low (dark) force regions. The individual force–distance curves *F*(*z*) included in the map are shown in the left panel. In both acidic and basic solutions (pH 1–13), the interlayer spacing is ∼0.28 nm (Figure 2a–g). This value lied within the 0.30 ± 0.03 nm range established by 3D-AFM, X-ray reflectivity, and molecular dynamics simulations for a hydration layer.^11^ The data did not show any dependence of the interlayer distance on the value of the pH.

**Figure 2:**
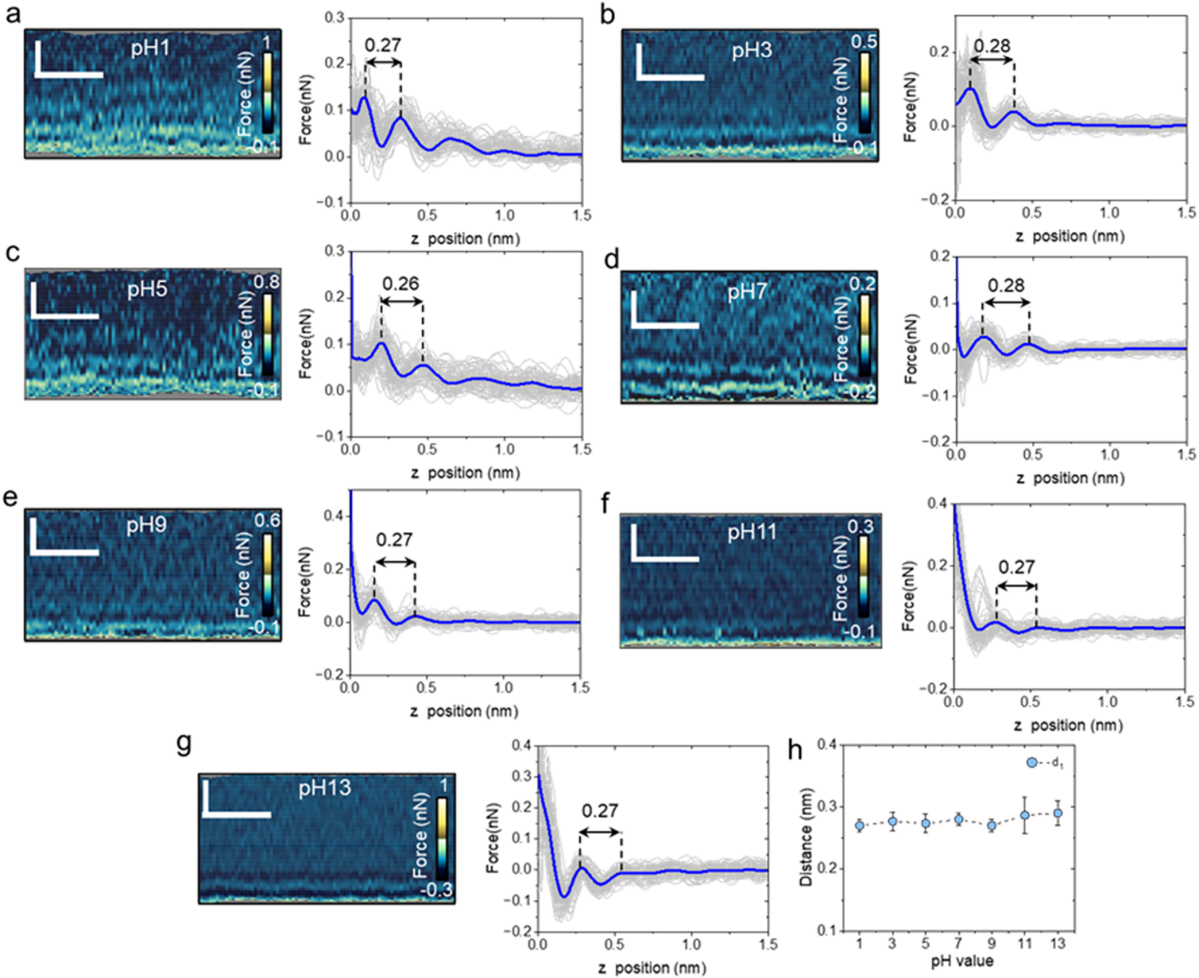
Interfacial water layer structure on mica as a function of the pH. (a–g) Representative 2D-AFM (*x*, *z*) force maps and corresponding force–distance curves of mica in aqueous solutions at pH values ranging from 1 to 13. The average force–distance curve is highlighted in blue, while individual force–distance curves are plotted in gray. The distances marked in the force–distance panels have units of nm. (h) Average value of the interlayer distance. The panel shows the average values obtained from three experiments similar to the one depicted in parts (a–g). Scale bars, 1 nm (horizontal) and 0.5 nm (vertical). 3D-AFM operational parameters are found in Table S1 (Supporting Information).

Figure 3 shows several force maps *F*(*x*, *z*) acquired on MoS_2_ surfaces at different pH values, alongside the corresponding force–distance curves. The force maps revealed a layered structure that extended about 1 nm from the MoS_2_ surface. The corresponding individual force–distance curves are shown in the left panel. These curves exhibited an oscillatory profile, from which we determined the interlayer distances. At the MoS_2_–water interface, *d*_1_ was in the 0.42–0.50 nm range (Figure 3h). Interlayer distance values above 0.40 nm were associated with the formation of hydrocarbon layers. The diameter of a linear alkane molecule is ∼0.42 nm. The data showed that *d*_1_ peaked at pH = 7 (*d*_1_ = 0.50 nm).

**Figure 3:**
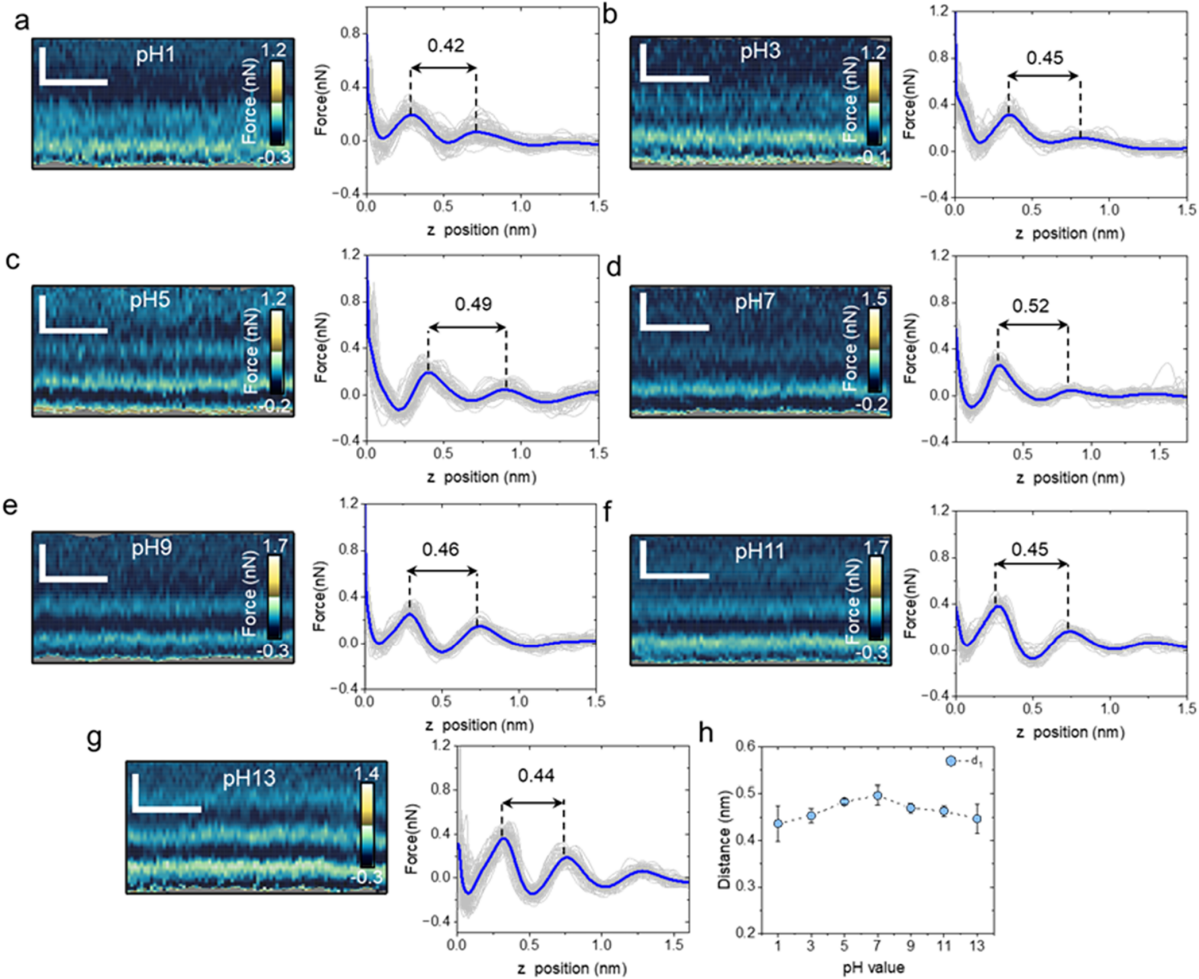
Interfacial water layer structure on MoS_2_ as a function of the pH. (a–g) 2D force maps (*x*, *z*) at a fixed *y* position and force–distance curves as a function of pH. The average force–distance curve is highlighted in blue, while individual force–distance curves are plotted in gray. The distances marked in the force–distance panels have units of nm. (h) Average value of the interlayer distance. The panel shows the average values obtained from three experiments similar to the one depicted in (a–g). Scale bars, 1 nm (horizontal) and 0.5 nm (vertical). 3D-AFM operational parameters are found in Table S1 (Supporting Information).

A similar experiment was performed on graphite (HOPG). Figure 4 shows the existence of two to three solvation layers. In some cases, the layer structure reached 1.5 nm from the graphite surface. This result underlined the strong affinity of the hydrocarbons for the graphite surface. For all of the pH values, *d*_1_ was in the 0.42–0.45 nm range (Figure 4h). The data showed that *d*_1_ peaked at pH = 7 (*d*_1_ = 0.45 nm).

**Figure 4:**
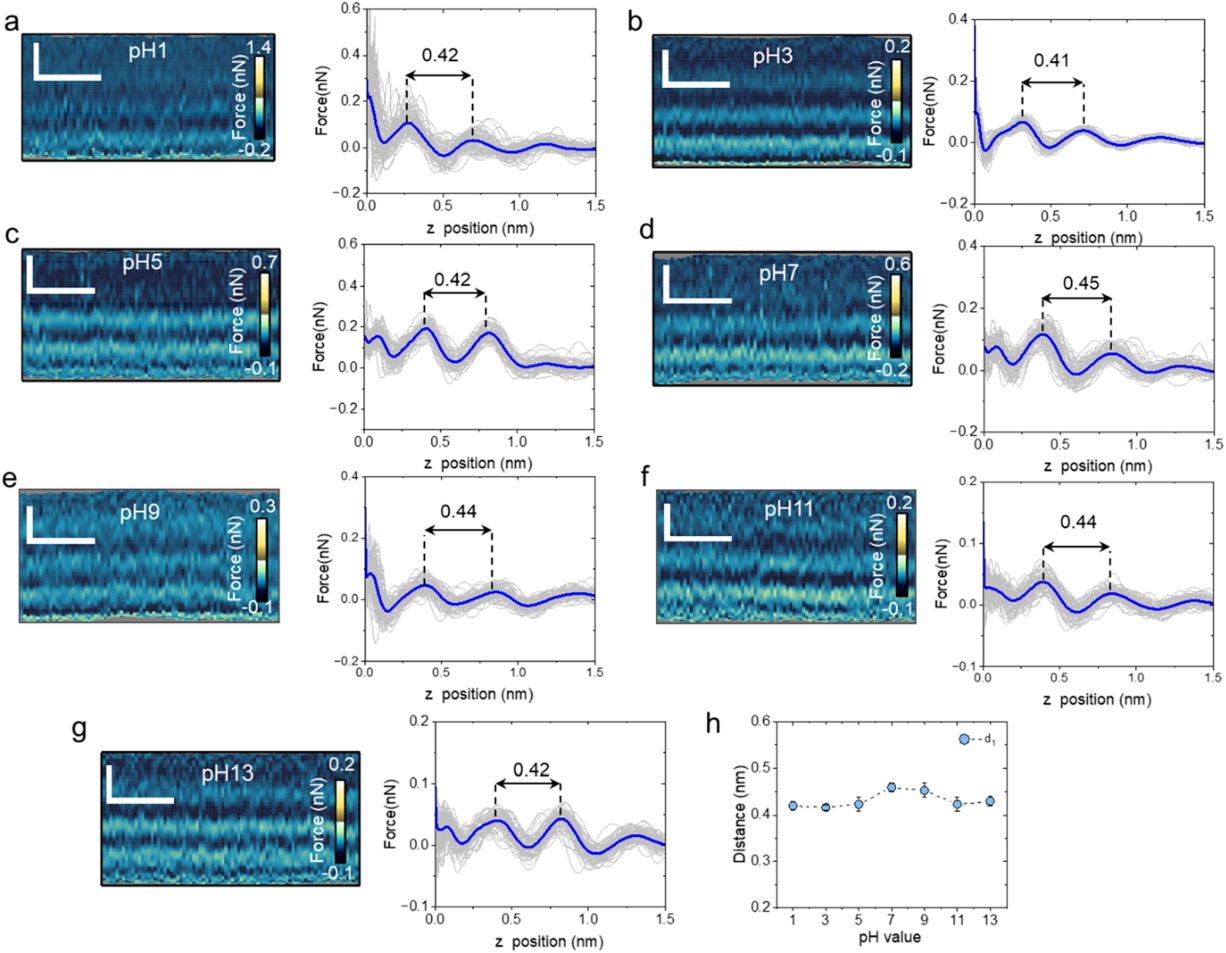
Interfacial water layer structure on graphite as a function of the pH. (a–g) 2D force maps (*x*, *z*) and force–distance curves as a function of pH. The average force–distance curve is highlighted in blue, while individual force–distance curves are plotted in gray. The distances marked in the force–distance panels have units of nm. (h). Average value of the interlayer distance. The panel shows the average values obtained from three experiments similar to the one depicted in parts (a–g). Scale bars, 1 nm (horizontal) and 0.5 nm (vertical). 3D-AFM operational parameters are found in Table S1 (Supporting Information).

The value of the force peaks might show some minor variations with pH (Figures 3 and 4). Those changes might arise from a variety of factors. Notably, the interfacial hydration structure of the tip’s apex might be modified by the pH. This effect will modify the effective tip’s size and the values of the force measured by the tip. However, those effects will not affect the determination of the interlayer distance values.

Figure 5 compares the dependence of *d*_1_ on the pH for MoS_2_, HOPG, and mica. On the van der Waals solids, MoS_2_ and HOPG, *d*_1_ values were in the 0.42–0.50 nm range, which is in agreement with the expected interlayer distance for layers made of a combination of straight-chain alkanes. The values were slightly smaller on HOPG (∼0.43 nm) than on MoS_2_ (∼0.47 nm). This difference was attributed to the higher affinity of the hydrocarbons for graphite. In contrast, on mica surfaces, *d*_1_ values were centered at 0.28 nm, which is in agreement with the distance separating hydration layers. Those differences underlined that on mica, the interfacial layer structure was made of water molecules organized in hydration layers, while on MoS_2_ and HOPG, the layers were made of straight-chain alkanes organized in hydrocarbon layers. Notably, neither strong acid nor base solutions affected the interlayer solvation structure observed on mica, MoS_2_, or HOPG. The persistence of interlayer distances of 0.45 nm highlighted that the charge of the solution had no effect on the adsorption of hydrocarbons on the van der Waals materials.

**Figure 5:**
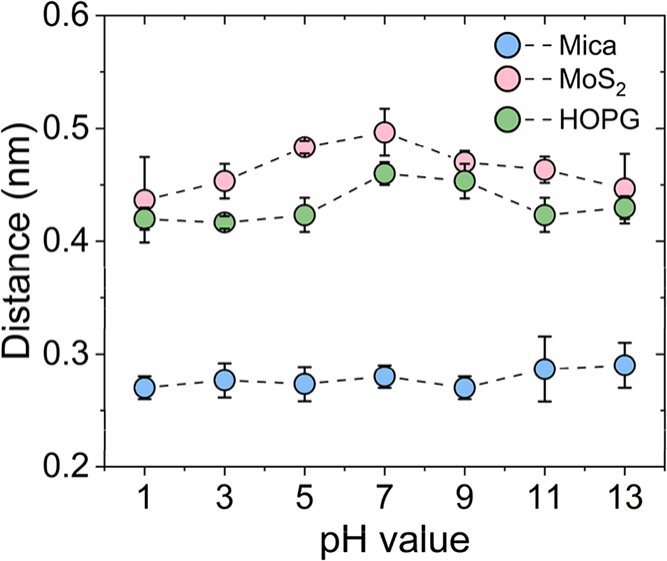
Interfacial layer spacing on different surfaces as a function of the pH. (a) MoS_2_, (b) HOPG, and (c) mica. Data points represent the mean values of three independent experiments. Error bars denote standard deviation (SD) values.

The ion concentration (molarity) changed with the pH, with the highest value, 100 mM, at pH 1 and 13 (Table S1). Those concentrations did not modify the periodicity of the hydration layers on mica because the concentration of water molecules outnumbered those of the ions. This observation agreed with theory^46^ and other experiments.^42^

### 2.2 Free Energy Considerations

The concentration of linear alkanes in air and ultrapure water is very small, respectively, ∼20 μg/m^3^ ^51,52^ and ∼3 μg/L (3 ppb). For that reason, the prevalence of hydrocarbon layers on van der Waals material–water interfaces might be surprising. The existence of a hydrocarbon layer at the interface of crystalline hydrophobic surfaces has been demonstrated theoretically by using free energy considerations.^24^ In addition, molecular dynamics simulations have shown that the organic molecules have a high affinity to be adsorbed on graphite-like surfaces from aqueous solutions.^53–55^

The adsorption of hydrocarbons on a van der Waals surface immersed in water can be determined from the effective Hamiltonian^24^

1 $${H=\sum_{i}\left({\Delta G}_{\mathrm{air}\to \mathrm{water}}+\Delta G_{\mathrm{water}\to \mathrm{ads}}-k_{B}T\ln\left(c/c_{0}\right)+\left\langle \sigma \right\rangle {\Delta G}_{\mathrm{ads}\to \mathrm{layer}}\right)\sigma }_{i}$$

where Δ*G*_air→water_ is the hydration free energy of a single hydrocarbon molecule; Δ*G*_water→ads_ is the free energy of adsorption of an isolated molecule from the water phase; Δ*G*_ads→layer_ is the free energy of transferring an adsorbed molecule to the interfacial layer phase; hence, Δ*G*_ads→layer_ < 0 represents the adsorbate–adsorbate attraction; *c* is the ambient concentration in air; *c*_0_ is the mass density of the interfacial hydrocarbon layer phase, which can be approximated by the mass density of the alkane; μ = *k*_B_ *T* ln­(*c*/*c*_0_) is the chemical potential of hydrocarbon molecules; σ_i_ are the interfacial sites that can be occupied or unoccupied by single hydrocarbon molecules with σ*i* ∈ {0, 1}; and ⟨σ⟩ is the mean occupancy of surface sites, which is calculated by

2 $$\left\langle \sigma \right\rangle ={\left\{1+\left(c_{0}/c\right)\exp\left[{\left(k_{B}T\right)}^{-1}\left(\Delta G_{\mathrm{air}\to \mathrm{water}}+\Delta G_{\mathrm{water}\to \mathrm{ads}}+\left\langle \sigma \right\rangle \Delta G_{\mathrm{ads}\to \mathrm{layer}}\right)\right]\right\}}^{-1}$$

Equation 2 says that above a critical concentration, there is a high-coverage solution which marks the transition from adsorption of dilute, isolated molecules to nucleation and growth of a nearly complete layer.^24^ We proceeded to determine the critical concentration to obtain a monolayer for a straight-chain alkane with 18 carbons (C18) by using *c*_0_ = 0.75 g/mL, Δ*G*_air→water_ = +5.02 kcal/mol, Δ*G*_water→ads_ = −14.34 kcal/mol, and Δ*G*_ads→layer_ = −9.23 kcal/mol. We used the free energy values reported in ref 54. The presence of C18 alkanes was compatible with 3D-AFM data^23,24^ and MD simulations.^54^ Furthermore, they might be considered as representative of the straight-chain alkanes detected in airborne organic contaminants.^51,52^

By introducing those values in eq 2, we deduced a critical concentration for the formation of a hydrocarbon layer of 15 μg/m^3^. That value was comparable to or even smaller than the concentration of airborne organic contaminants (∼20 μg/m^3^). The model showed that the displacement of water by hydrocarbons and the formation of hydrocarbon layers were driven by the hydrophobic interaction and the presence of hydrocarbons in the environment. Therefore, the observation of hydrocarbon layers in the 3D-AFM images was fully explained by theory.

The above model also explained a puzzling observation. The adsorption and formation hydrocarbon layers on van der Waals materials was more pronounced in water than in an air environment.^11,56^ This observation might be explained by the hydrophobic effect, which caused both Δ*G*_water→ads_ and Δ*G*_ads→layer_ to be negative. Therefore, a van der Waals surface immersed in water will act as an attractor of the alkane molecules that might be present in the solution.

### 2.3 Implications on the Properties of 2D Material–Water Interfaces

The above findings demonstrated the stability and prevalence of hydrocarbon layers on graphite and MoS_2_ for acidic and alkaline conditions. This behavior seemed to capture a universal property of the interaction of liquid water with an extended hydrophobic surface under standard working conditions. Many experimental results involving van der Waals material–water interfaces^15,16,18,25–27,57,58^ might be reinterpreted in terms of the presence of hydrocarbon layers.

#### 2.3.1 Moisture Stability Monolayer MoS_2_ and MoSe_2_

Choi et al.^26^ showed that monolayer transition metal dichalcogenides grown on hydrophobic surfaces retained stable structural and electronic properties at a very high relative humidity. They reported an experiment where monolayers (ML) were grown, respectively, on hydrophilic (silica) and hydrophobic (ODTS) surfaces. Both samples were immersed in a solution for about 3 h. The monolayers grown on the hydrophilic surface showed a quick degradation, while the ones grown on the hydrophobic surface retained their structural integrity. The paper does not provide an explanation. However, that result could be readily explained in terms of the hydrophobic layers. In both cases, the interaction of the ML with the aqueous solution was dominated by the van der Waals interaction with the substrate (wetting transparency effect^59^). On the hydrophobic substrate, the van der Waals interaction of the substrate with the solution favored the formation of hydrophobic layers on the ML. Those layers passivated the surface and prevented its degradation. In contrast, on the ML grown on the hydrophilic substrate, the van der Waals interaction favored the adsorption of water molecules and the subsequent formation of hydrophilic layers. The water molecules within the hydration layers were free to react with defects in the ML, which eventually led to degradation of the whole monolayer.

#### 2.3.2 Low Dielectric Constant of Confined Water

It has been hypothesized that interfacial water molecules exhibit electric properties that can be drastically different from those of bulk water. A scanning dielectric microscopy experiment measured the capacitance of a nanofluidic channel filled with liquid water as a function of the separation between the top and bottom 2D-crystal surfaces. The data showed that the effective dielectric constant of the interface decreased from 80 at large separations to ∼2 at 1 nm. Fumagalli et al. proposed that a value of ε = 2 was caused by a strong interaction happening between the water molecules and the 2D-crystal surface.^27^ This interaction restricted the rotational degrees of freedom of water molecules, which led to the decrease in ε. However, the presence of hydrocarbon layers at the 2D-crystal surfaces provided an alternative explanation. Figures 3 and 4 showed that a nanofluidic channel of 1 nm should be filled with two or three hydrocarbon layers. Based on the dielectric constant value of alkane molecules at *T* = 295 K (ε ≈ 2), the resulting dielectric constant of the nanofluid channel should be precisely 2. Therefore, for a nanofluidic channel of height about 1 nm, the dielectric constant might be dominated by the dielectric properties of alkane molecules instead of water.

#### 2.3.3 Controllable van der Waals Gaps by Water Adsorption

Liu et al. developed a technique to control the height of van der Waals (vdW) gaps between two-dimensional (2D) crystals.^58^ The technique is based on the preadsorption of water molecules on a MoS_2_ surface. Then, this MoS_2_ surface was laminated to another MoS_2_ surface, and the preadsorbed water was squeezed and confined between the flat upper/lower surfaces. This last step gave rise to a vdW gap filled with a water film. The minimum gap obtained by Liu et al. was 5.5 Å. This value is 2-fold larger than the diameter of a water molecule (2.8 Å). Therefore, the gap suggests the presence of two hydration layers. Alternatively, the gap could have been generated by the adsorption of a monolayer of alkane molecules. The latter possibility would be consistent with our observations.

## 3 Conclusion

We have studied the interfacial liquid layer structure on two van der Waals materials (graphite and MoS_2_) and mica (hydrophilic) from acidic to alkaline pH values. On mica, angstrom-scale resolution 3D-AFM images revealed the presence of 2–3 hydration layers with an interlayer distance of 0.28 nm. That value did not depend on the pH value. It remained practically unchanged for experiments lasting up to 2 h.

On the other hand, angstrom-scale resolution images obtained on MoS_2_ and graphite-water interfaces prepared under standard working conditions showed the presence of 2–3 hydrocarbon layers separated by 0.45 nm. Those layers were associated with the adsorption of straight-chain alkanes. Hydrocarbons expelled water molecules from the interface. The 3D-AFM images showed that the pH of the solution neither prevented the presence nor favored the removal of hydrocarbon layers from the vdW material–water interface.

These findings provided the most accurate description of the influence of pH on the interfacial water layer structure on hydrophilic (mica) and mildly hydrophobic (van der Waals materials) surfaces. We found that the pH has little influence on the interfacial layer structure on mica, graphite, and MoS_2_ surfaces. A theoretical model based on free energy considerations supported the experimental observations. The findings presented above might be extended to other crystalline hydrophobic surfaces. On those surfaces, hydrocarbon molecules, not water, will dictate the interfacial layer spacing with independence of the concentrations of protons or hydroxide ions. These findings indicated that hydrocarbon layers should be explicitly considered to explain the properties of van der Waals material–water interfaces.

## 4 Experimental Methods

### 4.1 3D-AFM

The microscope has two distinctive units: the AFM platform and the unit to control the tip’s displacements. The AFM platform was a Cypher S (Asylum Research). The home-built unit was developed to synchronize the *xyz* tip’s displacements. A 2.0 nm peak-to-peak sinusoidal modulation at a frequency of 100 Hz (period of 10 ms) was superimposed on the *z*-piezo, and the *xy* scan path was adapted so that data were collected on both forward and reverse passes. The *z*-piezo signal was synchronized with the *xy* motion to execute one complete *z*-cycle at every *xy* position. 3D-AFM was performed in the amplitude-modulation mode^60^ with the cantilever driven at its first eigenmode by photothermal excitation at 405 nm. The free oscillation amplitude *A*_0_ was in the 60–250 pm range, and set-point values were *A*_sp_ = 0.5–0.90 *A*_0_. See specific details in Table S1 (Supporting Information).

A feedback bandwidth of 2 kHz enabled tracking of the surface topography and correct for the sample tilt while minimizing coupling with the *z* modulation. The *z*-channel was sampled every 10.24 μs and stored at 512 points (256 per half-cycle). Each 2D force map had 80 × 64 pixels, which implied a total acquisition time of 52 s for a volume image. Unless otherwise specified, the experiment began by acquiring the first image after 10 min of placing the sample in the fluid cell.

Force–distance curves were reconstructed from amplitude and phase versus distance data. An exponential function was subtracted to remove long-range contributions (nonoscillatory).^22^

Sharpened silicon cantilevers (ArrowUHF AuD, NanoAndMore, Germany) were used for the 3D-AFM imaging. See specific details in Table S1 (Supporting Information). New cantilevers were used as received. Reused cantilevers were sequentially cleaned in deionized water, acetone, isopropanol, and ethanol and dried with nitrogen before loading them in the 3D-AFM. In air, each cantilever was thermally calibrated with GetReal software to determine the spring constant, quality factor *Q*_1_, and resonant frequency *f*_1_.^61^

### 4.2 Materials

HOPG, MoS_2_, and mica were obtained from commercial sources. We fixed the bulk specimen to a PTFE sample disk with an epoxy adhesive. Highly oriented pyrolytic graphite (HOPG, grade ZYB) and MoS_2_ crystals were purchased from HQ Graphene (The Netherlands). The HOPG was freshly cleaved with 3 M Magic tape immediately for imaging. Muscovite mica (grade V-1; SPI Supplies) was cleaved in the same manner and promptly immersed in the solution.

### 4.3 Solvents

Deionized water (18.2 MΩ cm^–1^; HI 9024, Hanna Instruments) was freshly produced for each experiment. The starting pH was 5.6. Acidic solutions (pH 1–5) were obtained by dropwise addition of concentrated H_2_SO_4_ (96%, PA). Basic solutions (pH 7–13) were prepared by serial dilution of a 1 M KOH stock solution made from KOH powder (85%, Sigma-Aldrich). Target pH values spanned 1, 3, 5, 7, 9, 11, and 13 and were verified with a calibrated meter (Hanna HI9024). Approximately 30 μL of the corresponding solution was dispensed onto each substrate with a micropipette. A 50 mM KCl electrolyte was prepared by dissolving KCl powder (99%, Sigma-Aldrich) in DI water (pH ≈ 5.6).

## Supplementary Material



## Notes

Tables S1 and S2 summarize, respectively, the cantilever parameters used in the 3D-AFM experiments and the concentration of the relevant ions for the different pH values (PDF)

## References

[1] Chen X., McCrum I. T., Schwarz K. A., Janik M. J., Koper M. T. M. (2017). Co-adsorption
of Cations as the Cause of the Apparent pH Dependence of Hydrogen
Adsorption on a Stepped Platinum Single-Crystal Electrode. Angew. Chem., Int. Ed..

[2] Cui W. G., Gao F., Na G., Wang X., Li Z., Yang Y., Niu Z., Qu Y., Wang D., Pan H. (2024). Insights into the pH
effect on hydrogen electrocatalysis. Chem. Soc.
Rev..

[3] Garcia-Sacristan C., Gisbert V. G., Klein K., Saric A., Garcia R. (2024). In Operando
Imaging Electrostatic-Driven Disassembly and Reassembly of Collagen
Nanostructures. ACS Nano.

[4] Dewan S., Yeganeh M. S., Borguet E. (2013). Experimental
Correlation Between
Interfacial Water Structure and Mineral Reactivity. J. Phys. Chem. Lett..

[5] Philip A., Kumar A. R. (2023). Recent advancements
and developments employing 2D-materials
in enhancing the performance of electrochemical supercapacitors: A
review. Renewable Sustainable Energy Rev..

[6] Wang Z., Tu Q., Zheng S., Urban J. J., Li S., Mi B. (2017). Understanding
the Aqueous Stability and Filtration Capability of MoS(2) Membranes. Nano Lett..

[7] Takahashi Y., Kobayashi Y., Wang Z., Ito Y., Ota M., Ida H., Kumatani A., Miyazawa K., Fujita T., Shiku H., Korchev Y. E., Miyata Y., Fukuma T., Chen M., Matsue T. (2020). High-Resolution Electrochemical Mapping of the Hydrogen
Evolution Reaction on Transition-Metal Dichalcogenide Nanosheets. Angew. Chem., Int. Ed..

[8] Zhang D., Li Z., Klausen L. H., Li Q., Dong M. (2022). Friction behaviors
of two-dimensional materials at the nanoscale. Mater. Today Phys..

[9] Velasco A., Ryu Y. K., Boscá A., Ladrón-de-Guevara A., Hunt E., Zuo J., Pedrós J., Calle F., Martinez J. (2021). Recent trends in graphene
supercapacitors:
from large area to microsupercapacitors. Sustainable
Energy Fuels.

[10] Gao Q., Tsai W. Y., Balke N. (2021). In situ and operando force-based
atomic force microscopy for probing local functionality in energy
storage materials. Electrochem. Sci. Adv..

[11] Garcia R. (2023). Interfacial
Liquid Water on Graphite, Graphene, and 2D Materials. ACS Nano.

[12] Kozbial A., Gong X., Liu H., Li L. (2015). Understanding the Intrinsic
Water Wettability of Molybdenum Disulfide (MoS2). Langmuir.

[13] Chen X., Yang Z., Feng S., Golbek T. W., Xu W., Butt H. J., Weidner T., Xu Z., Hao J., Wang Z. (2020). How Universal Is the Wetting Aging in 2D Materials. Nano Lett..

[14] Gallagher P., Lee M., Amet F., Maksymovych P., Wang J., Wang S., Lu X., Zhang G., Watanabe K., Taniguchi T., Goldhaber-Gordon D. (2016). Switchable friction enabled by nanoscale self-assembly
on graphene. Nat. Commun..

[15] Yang C. W., Miyazawa K., Fukuma T., Miyata K., Hwang I. S. (2018). Direct
comparison between subnanometer hydration structures on hydrophilic
and hydrophobic surfaces via three-dimensional scanning force microscopy. Phys. Chem. Chem. Phys..

[16] Lu Y. H., Yang C. W., Hwang I. S. (2012). Molecular
layer of gaslike domains
at a hydrophobic-water interface observed by frequency-modulation
atomic force microscopy. Langmuir.

[17] Wastl D. S., Speck F., Wutscher E., Ostler M., Seyller T., Giessibl F. J. (2013). Observation of 4
nm pitch stripe domains formed by
exposing graphene to ambient air. ACS Nano.

[18] Schlesinger I., Sivan U. (2018). Three-Dimensional
Characterization of Layers of Condensed Gas Molecules
Forming Universally on Hydrophobic Surfaces. J. Am. Chem. Soc..

[19] Temiryazev A., Frolov A., Temiryazeva M. (2019). Atomic-force
microscopy study of
self-assembled atmospheric contamination on graphene and graphite
surfaces. Carbon.

[20] Seibert S., Klassen S., Latus A., Bechstein R., Kuhnle A. (2020). Origin of Ubiquitous Stripes at the
Graphite-Water
Interface. Langmuir.

[21] Eichhorn A. L., Hoffer M., Dietz C. (2022). In-plane and
out-of-plane interaction
analysis of adsorbates on multilayer graphene and graphite by multifrequency
atomic force microscopy. Carbon.

[22] Uhlig M. R., Martin-Jimenez D., Garcia R. (2019). Atomic-scale mapping of hydrophobic
layers on graphene and few-layer MoS_2_ and WSe_2_ in water. Nat. Commun..

[23] Uhlig M. R., Benaglia S., Thakkar R., Comer J., Garcia R. (2021). Atomically
resolved interfacial water structures on crystalline hydrophilic and
hydrophobic surfaces. Nanoscale.

[24] Arvelo D.
M., Comer J., Schmit J., Garcia R. (2024). Interfacial Water Is
Separated from a Hydrophobic Silica Surface by a Gap of 1.2 nm. ACS Nano.

[25] Duignan T. T., Zhao X. S. (2019). Impurities Limit
the Capacitance of Carbon-Based Supercapacitors. J. Phys. Chem. C.

[26] Choi S. Y., Kim S., Kim Y., Park M., Son M. G., Ma J. Y., Park Y. M., Kim J. H., Kang H., Kim J., Lee W. B., Seo B., Kim H. H. (2025). Wetting Transparency-Induced
Enhancement of Moisture Stability in Monolayer Transition Metal Dichalcogenides. Small.

[27] Fumagalli L., Esfandiar A., Fabregas R., Hu S., Ares P., Janardanan A., Yang Q., Radha B., Taniguchi T., Watanabe K., Gomila G., Novoselov K. S., Geim A. K. (2018). Anomalously low dielectric constant of confined water. Science.

[28] Aluru N. R., Aydin F., Bazant M. Z., Blankschtein D., Brozena A. H., de Souza J. P., Elimelech M., Faucher S., Fourkas J. T., Koman V. B., Kuehne M., Kulik H. J., Li H. K., Li Y., Li Z., Majumdar A., Martis J., Misra R. P., Noy A., Pham T. A., Qu H., Rayabharam A., Reed M. A., Ritt C. L., Schwegler E., Siwy Z., Strano M. S., Wang Y., Yao Y. C., Zhan C., Zhang Z. (2023). Fluids and Electrolytes under Confinement
in Single-Digit Nanopores. Chem. Rev..

[29] Arvelo D. M., Garcia R. (2025). Molecular-Scale Resolution Mapping of Electrified MoS2–Water
Interfaces by Operando 3D-AFM. ACS Electrochem..

[30] Bonagiri L. K. S., Arvelo D. M., Zhao F., Kim J., Ai Q., Zhou S., Panse K. S., Garcia R., Zhang Y. J. (2026). Probing
the molecular structure at graphite–water interfaces by correlating
3D-AFM and SHINERS. Nat. Commun..

[31] Fukuma T., Garcia R. (2018). Atomic- and Molecular-Resolution
Mapping of Solid-Liquid
Interfaces by 3D Atomic Force Microscopy. ACS
Nano.

[32] Fukuma T., Ueda Y., Yoshioka S., Asakawa H. (2010). Atomic-scale distribution
of water molecules at the mica-water interface visualized by three-dimensional
scanning force microscopy. Phys. Rev. Lett..

[33] Kimura K., Ido S., Oyabu N., Kobayashi K., Hirata Y., Imai T., Yamada H. (2010). Visualizing water molecule distribution by atomic force
microscopy. J. Chem. Phys..

[34] Fukuma T., Reischl B., Kobayashi N., Spijker P., Canova F. F., Miyazawa K., Foster A. S. (2015). Mechanism of atomic force microscopy
imaging of three-dimensional hydration structures at a solid-liquid
interface. Phys. Rev. B.

[35] Söngen H., Reischl B., Miyata K., Bechstein R., Raiteri P., Rohl A. L., Gale J. D., Fukuma T., Kühnle A. (2018). Resolving Point Defects in the Hydration
Structure
of Calcite (10.4) with Three-Dimensional Atomic Force Microscopy. Phys. Rev. Lett..

[36] Martin-Jimenez D., Chacon E., Tarazona P., Garcia R. (2016). Atomically resolved
three-dimensional structures of electrolyte aqueous solutions near
a solid surface. Nat. Commun..

[37] Martin-Jimenez D., Garcia R. (2017). Identification of Single Adsorbed Cations on Mica-Liquid
Interfaces by 3D Force Microscopy. J. Phys.
Chem. Lett..

[38] Nakouzi E., Stack A. G., Kerisit S., Legg B. A., Mundy C. J., Schenter G. K., Chun J., De Yoreo J. J. (2021). Moving
beyond the
Solvent-Tip Approximation to Determine Site-Specific Variations of
Interfacial Water Structure through 3D Force Microscopy. J. Phys. Chem. C.

[39] Bonagiri L. K. S., Panse K. S., Zhou S., Wu H., Aluru N. R., Zhang Y. (2022). Real-Space Charge Density Profiling
of Electrode-Electrolyte Interfaces
with Angstrom Depth Resolution. ACS Nano.

[40] Li Z., Liu Q., Zhang D., Wang Y., Zhang Y., Li Q., Dong M. (2022). Probing the hydration friction of ionic interfaces at the atomic
scale. Nanoscale Horiz..

[41] Li Z., Liu Q., Li Q., Dong M. (2023). The role of hydrated anions in hydration
lubrication. Nano Res..

[42] Nakouzi E., Kerisit S. N., Heo J., Legg B. A., Sassi M., Simonnin P., Rosso K. M. (2025). Effect
of Ions on Solution Structure
and Hydration Forces at the Orthoclase–Water Interface. J. Phys. Chem. C.

[43] van
Lin S. R., Grotz K. K., Siretanu I., Schwierz N., Mugele F. (2019). Ion-Specific and pH-Dependent Hydration of Mica-Electrolyte
Interfaces. Langmuir.

[44] Wang J., Li H., Tavakol M., Serva A., Nener B., Parish G., Salanne M., Warr G. G., Voitchovsky K., Atkin R. (2024). Ions Adsorbed at Amorphous
Solid/Solution Interfaces Form Wigner
Crystal-like Structures. ACS Nano.

[45] Miyazawa K., Kobayashi N., Watkins M., Shluger A. L., Amano K., Fukuma T. (2016). A relationship between three-dimensional surface hydration
structures and force distribution measured by atomic force microscopy. Nanoscale.

[46] Benaglia S., Uhlig M. R., Hernández-Muñoz J., Chacón E., Tarazona P., Garcia R. (2021). Tip Charge Dependence
of Three-Dimensional AFM Mapping of Concentrated Ionic Solutions. Phys. Rev. Lett..

[47] Hashimoto K., Amano K. I., Nishi N., Onishi H., Sakka T. (2021). Comparison
of atomic force microscopy force curve and solvation structure studied
by integral equation theory. J. Chem. Phys..

[48] Ai Q., Bonagiri L. K. S., Farokh
Payam A., Aluru N. R., Zhang Y. (2025). Toward Quantitative
Interpretation of 3D Atomic Force Microscopy at Solid–Liquid
Interfaces. J. Phys. Chem. C.

[49] Ricci M., Spijker P., Voitchovsky K. (2014). Water-induced
correlation between
single ions imaged at the solid-liquid interface. Nat. Commun..

[50] Trewby W., Tavakol M., Jaques Y. M., Voïtchovsky K. (2024). Towards local
tracking of solvated metal ions at solid-liquid interfaces. Mater. Today Phys..

[51] Brown S. K., Sim M. R., Abramson M. J., Gray C. N. (1994). Concentrations of
Volatile Organic Compounds in Indoor Air - A Review. Indoor Air.

[52] Halios C. H., Landeg-Cox C., Lowther S. D., Middleton A., Marczylo T., Dimitroulopoulou S. (2022). Chemicals
in European residences
- Part I: A review of emissions, concentrations and health effects
of volatile organic compounds (VOCs). Sci. Total
Environ..

[53] Azhagiya
Singam E. R., Zhang Y., Magnin G., Miranda-Carvajal I., Coates L., Thakkar R., Poblete H., Comer J. (2019). Thermodynamics
of Adsorption on Graphenic Surfaces from Aqueous Solution. J. Chem. Theory Comput..

[54] Thakkar R., Gajaweera S., Comer J. (2022). Organic contaminants and atmospheric
nitrogen at the graphene-water interface: a simulation study. Nanoscale Adv..

[55] Uematsu Y., Iwai S., Konishi M., Inagi S. (2024). Zeta Potentials of
Cotton Membranes in Acetonitrile Solutions. Langmuir.

[56] Haghighian N., Convertino D., Miseikis V., Bisio F., Morgante A., Coletti C., Canepa M., Cavalleri O. (2018). Rippling of
graphitic surfaces: a comparison between few-layer graphene and HOPG. Phys. Chem. Chem. Phys..

[57] Utsunomiya T., Yokota Y., Enoki T., Fukui K. (2014). Potential-dependent
hydration structures at aqueous solution/graphite interfaces by electrochemical
frequency modulation atomic force microscopy. Chem. Commun..

[58] Liu C., Zou X., Lv Y., Liu X., Ma C., Li K., Liu Y., Chai Y., Liao L., He J. (2024). Controllable van der
Waals gaps by water adsorption. Nat. Nanotechnol..

[59] Belyaeva L. A., Schneider G. F. (2020). Wettability of graphene. Surf.
Sci. Rep..

[60] Garcia R., San Paulo A. (1999). Attractive and repulsive tip-sample
interaction regimes
in tapping-mode AFM. Phys. Rev. B.

[61] Labuda A., Kocun M., Lysy M., Walsh T., Meinhold J., Proksch T., Meinhold W., Anderson C., Proksch R. (2016). Calibration
of higher eigenmodes of cantilevers. Rev. Sci.
Instrum..

