# Supplementary material for: Angstrom-Scale Water Layer Structure on van der Waals Materials Probed by 3D Atomic Force Microscopy: From Acidic to Alkaline Aqueous Solutions
Source: Langmuir. 2026 Feb 10;42(7):5608–16. doi: 10.1021/acs.langmuir.5c05798 (PMC12937111; doi:10.1021/acs.langmuir.5c05798)
Supplement: Supplementary file 1 [file la5c05798_si_001.pdf]

Supporting information

Angstrom-scale water layer structure on van der Waals materials probed by 3D atomic force microscopy: From acidic to alkaline aqueous solutions

*Zhen Tang and Ricardo Garcia**

Instituto de Ciencia de Materiales de Madrid, CSIC, 28049 Madrid, Spain.

Email: r.garcia@csic.es

Contains

Table S1: Cantilever-tip parameters
Table S2: Concentrations

Table S1. Cantilever parameters in liquid used for 3D-AFM measurements.

Table S1 Tip parameter									
Sample	Figure	Figure	Type	k (N/m)	Q	f(kHz)	inoVLS (nm/V)	A_0	A_{sp}
Mica	Fig.2	a,c	ArrowUHF AuD	22.8	6.4	1077	9.65	96.5	77.2
		b,d		13.32	4.9	672.8	10.5	63	52.5
		e,f,g		13.76	5.4	633.1	13.73	68.65	54.92
		h		15.22	6.4	694.2	12.57	50.28	43.995
MOS_2	Fig.3	a-d		13.38	6.2	803.447	9.71	165.07	126.23
		e-f		13.8	6.2	913.346	9.37	196.77	149.92
		g		11.86	5.1	622.041	12.26	196.16	183.9
		h		12.4	6	715.69	13.11	235.98	196.65
HOPG	Fig.4	a,g,f,h		17.63	6.2	733.979	15.54	186.48	124.32
		d,e		10.17	5.1	560.81	12.36	160.68	86.52
		b,c		16.9	5.9	732.112	17.49	174.9	104.94

Table S2. Calculated concentrations of relevant ions in solutions at different pH values.

Table S2.Calculated concentrations of relevant ions in solutions at different pH values.						
	pH	$[H^+]$ (M)	$[OH^-]$ (M)	$[HSO_4^-]$ (M)	$[SO_4^{2-}]$ (M)	$[K^+]$ (M)
	1	1.00E-01	1.00E-13	8.06E-02	9.68E-03	—
	2	1.00E-02	1.00E-12	2.94E-03	3.53E-03	—
	3	1.00E-03	1.00E-11	4.00E-05	4.80E-04	—
	5	1.00E-05	1.00E-09	4.16E-09	5.00E-06	—
	7	1.00E-07	1.00E-07	—	—	1.00E-07
	9	1.00E-09	1.00E-05	—	—	1.00E-05
	11	1.00E-11	1.00E-03	—	—	1.00E-03
	13	1.00E-13	1.00E-01	—	—	1.00E-01
